# How to be an attractive male: floral dimorphism and attractiveness to pollinators in a dioecious plant

**DOI:** 10.1186/1471-2148-9-190

**Published:** 2009-08-06

**Authors:** Marc O Waelti, Paul A Page, Alex Widmer, Florian P Schiestl

**Affiliations:** 1 Plant Ecological Genetics, Institute of Integrative Biology, ETH Zürich, Universitätstrasse 16, 8092 Zürich, Switzerland; 2 Institute of Systematic Botany, University of Zürich, Zollikerstrasse 107, 8008 Zürich, Switzerland

## Abstract

**Background:**

Sexual selection theory predicts that males are limited in their reproductive success by access to mates, whereas females are more limited by resources. In animal-pollinated plants, attraction of pollinators and successful pollination is crucial for reproductive success. In dioecious plant species, males should thus be selected to increase their attractiveness to pollinators by investing more than females in floral traits that enhance pollinator visitation. We tested the prediction of higher attractiveness of male flowers in the dioecious, moth-pollinated herb *Silene latifolia*, by investigating floral signals (floral display and fragrance) and conducting behavioral experiments with the pollinator-moth, *Hadena bicruris*.

**Results:**

As found in previous studies, male plants produced more but smaller flowers. Male flowers, however, emitted significantly larger amounts of scent than female flowers, especially of the pollinator-attracting compounds. In behavioral tests we showed that naïve pollinator-moths preferred male over female flowers, but this preference was only significant for male moths.

**Conclusion:**

Our data suggest the evolution of dimorphic floral signals is shaped by sexual selection and pollinator preferences, causing sexual conflict in both plants and pollinators.

## Background

According to sexual selection theory, males compete with each other over access to females since the reproductive success of a male is limited by the number of females he can fertilize, whereas female reproductive success is limited by resources available for producing offspring [1,2]. In plants, access to pollinators should therefore limit the reproductive success of males to a greater extent than it restricts the reproductive success of females [3-5]. Consequently, different selection pressures are expected to act on males and females, resulting in male-male competition over mates [1].

The majority of plants rely on pollinators for successful pollen transfer [6]. Prefertilization-competition among male gametophytes has been described as pollen competition within the female organs [7], which can be influenced through physiological interactions with the pistils [8]. However, pollinator attraction is the first step in the reproductive cycle of animal-pollinated plants and in dioecious species, sexual reproduction is impossible without the transfer of pollen from male to female flowers. In contrast to most other plants that have hermaphroditic flowers, males and females can respond differently to pollinator-mediated selection. In this situation, selection may hence favor traits that improve pollination and fertilization success, which may lead to sexual dimorphisms in pollinator attracting traits rendering male flowers more attractive than females since access to mates is a function of access to pollinators [4,9,10]. In addition, natural selection on females should reduce attractiveness, since besides pollinators, floral signals also attract granivores that can drastically reduce fitness [11,12]. Even for male flowers, however, pollinator attraction is risky, as it may lead to infection by anther smut fungus that sterilizes the flowers [13].

It has been known for a long time that floral traits like color, shape, size and odor influence the behavior of flower visitors, but less is known about the relative importance of these traits in different pollination systems [14,15]. Colorful floral displays are often accompanied by fragrance, and both visual and olfactory signals attract pollinators and serve as learning cues [16]. In many nocturnal pollination systems, however, scent is thought to be of primary importance for pollinator attraction [16-18]. Floral signals in plants have a similar role in sexual reproduction as mating signals in animals, although they act indirectly, through the behavior of their pollinators. Thus, sexual dimorphisms in floral signals should evolve as a result of sexual selection, acting in concert with the neuronal and behavioral purge of the pollinators. Therefore, the signature of sexual selection should be especially pronounced in the signals that play a particular role in attraction of a given (guild of) pollinator(s).

We tested the prediction of higher attractiveness in male flowers by investigating floral signals and pollinator behavior in the perennial dioecious herb *Silene latifolia *that is primarily pollinated by nocturnal moths [11,19-21]. This species is an example of nursery pollination, as one of the main pollinators, the noctuid moth *Hadena bicruris*, oviposits in female flowers where larvae feed on developing seeds. We investigated flower size, flower number and floral odor emission in male and female *S. latifolia *plants. Further, we assessed the attractiveness of individual flowers of both sexes using male and female *Hadena bicruris *moths in a wind tunnel bioassay.

## Results

### Floral odor

**Figure 1:**
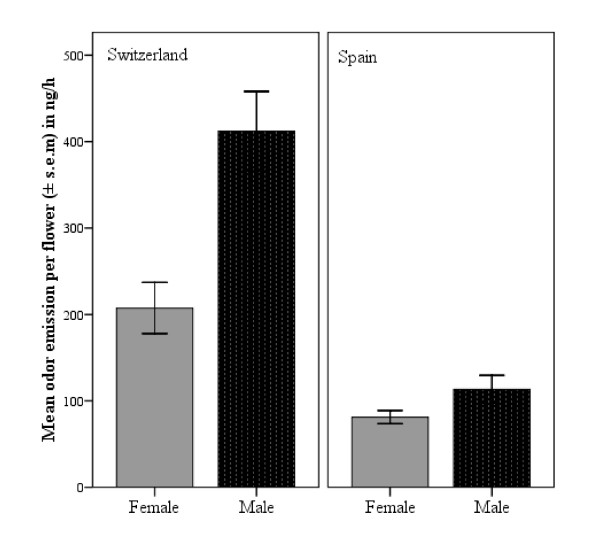
**Absolute amounts of odor emitted by female (grey bars) and male (black bars) flowers of two *S. latifolia *populations**. In both populations, male flowers emit significantly more odor than female flowers (GLM, P < 0.001).

In both populations investigated, male flowers produced significantly more odor than female flowers (Figure 1; Mean ± SE: Switzerland: males: 422.05 ± 47.34 ng h^-1^, females: 202.56 ± 25.57 ng h^-1^; Spain: males: 134.19 ± 16.88 ng h^-1^, females: 81.56 ± 7.90 ng h^-1^). In the GLM, both sex and population showed a significant effect and there was a significant interaction between sex and population (Table 1; GLM: sex*population: F_1,555 _= 11.252, P = 0.001), indicating that the Swiss population differed more strongly among sexes than did the Spanish population. The covariate flower diameter was not significant (Table 1).

**Table 1:** GLM of the effects of sex and population of *Silene latifolia *plants on log transformed total amount of odor per flower.

Source	Type III sum of squares	df	mean square	F	P
population	33.571	1	33.571	98.623	< 0.001
sex	12.560	1	12.560	36.899	< 0.001
population*sex	3.830	1	3.830	11.251	= 0.001
flower diameter	0.104	1	0.104	0.304	= 0.581

error	187.217	550	0.337		

**Figure 2:**
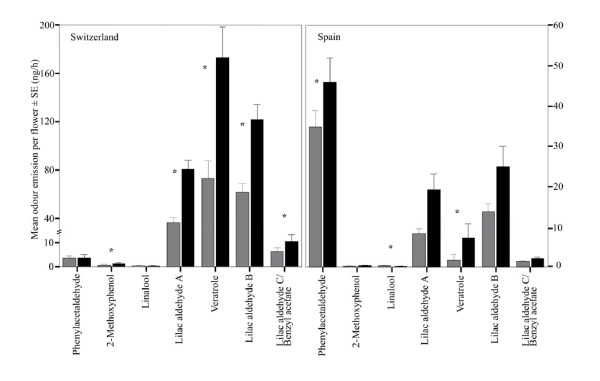
Mean emission of behaviorally active odor compounds by female (grey bars) and male (black bars) *S. latifolia *flowers in the Swiss (a) and the Spanish (b) population (Mann-Whitney U-test, * = p < 0.05). Note the difference in scale in the y-axes.

In the analysis of individual compounds, more active compounds (compounds triggering electrophysiological responses or affecting behavioral responses in pollinators) [17] were significantly different between the sexes than non-active compounds (Switzerland: active 93%, non-active: 50%; Spain active: 86%, non-active 62%). The emission of most compounds behaviorally active in *Hadena bicruris *[17] were found to be significantly higher in male flowers than in female flowers in both populations (Figure 2a, b, Table 2). In Switzerland, 2-methoxy phenol, the lilac aldehydes A, B and C, and veratrole were found in significantly higher amounts in male flowers. The amounts of phenylacetaldehyde and linalool were not significantly different between the sexes. In Spain, phenylacetaldehyde, lilac aldehyde A, and veratrole were found in significantly higher amounts in male flowers. 2-methoxy phenol, and the lilac aldehydes B and C were not significantly different in males and females, but showed a trend to higher emission in males. Only linalool was found in significantly higher amounts in females.

**Table 2:** Mean absolute amounts of odor compounds (± SEM; ng h^-1^) in headspace samples of *Silene latifolia *flowers (asterisks (*) indicate significant differences between female and male amounts within populations).

	**Switzerland**		**Spain**	
Compounds^1^	Females (N = 123) Mean ± SE	Males (N = 79) Mean ± SE	Females (N = 217) Mean ± SE	Males (N = 136) Mean ± SE
**Fatty Acid Derivates**^2^	**0.33%**	**0.13%**	**0.37%**	**0.17%**
Octanal	0.43 ± 0.14	0.33 ± 0.10	0.11 ± 0.01 *	0.10 ± 0.03
Nonanal^3^	0.24 ± 0.02	0.20 ± 0.03	0.19 ± 0.02 *	0.13 ± 0.02
				
**Aromatics**^2^	**41.41%**	**43.82%**	**57.58%**	**59.56%**
Benzaldehyde^3^	0.59 ± 0.08	0.48 ± 0.08	1.14 ± 0.18	0.94 ± 0.11
Phenylacetaldehyde^4^	3.49 ± 0.66	3.85 ± 1.53	34.80 ± 4.02 *	45.93 ± 6.07
2-Methoxy phenol^4^	0.68 ± 0.11 *	1.15 ± 0.21	0.08 ± 0.01	0.19 ± 0.04
Methyl benzoate^3^	0.04 ± 0.01 *	0.80 ± 0.46	0.06 ± 0.01 *	0.06 ± 0.01
2-Phenylethanol^3^	0.28 ± 0.13	0.31 ± 0.13	3.02 ± 0.54 *	2.26 ± 0.30
Veratrole^4^	72.94 ± 14.95 *	173.27 ± 25.43	1.88 ± 1.41 *	7.28 ± 3.53
Methyl salicylate^3^	0.85 ± 0.10 *	1.14 ± 0.34	5.41 ± 0.81 *	4.01 ± 0.84
Benzyl benzoate^3^	5.22 ± 3.20 *	3.95 ± 1.01	0.58 ± 0.11 *	0.34 ± 0.08
				
**Monoterpenes**^2^	**54.55%**	**52.67%**	**33.73%**	**37.24%**
α-Pinene	0.16 ± 0.01 *	0.12 ± 0.02	0.13 ± 0.01 *	0.08 ± 0.01
Camphene	0.16 ± 0.01	0.14 ± 0.02	0.12 ± 0.01 *	0.08 ± 0.01
β-Pinene	0.10 ± 0.01 *	0.06 ± 0.01	0.10 ± 0.01 *	0.06 ± 0.01
Limonene	0.46 ± 0.04	0.36 ± 0.03	0.36 ± 0.02 *	0.23 ± 0.02
Eucalyptol	0.52 ± 0.11 *	0.86 ± 0.17	0.76 ± 0.10	0.72 ± 0.11
Trans-β-Ocimene^3^	1.25 ± 0.53	1.95 ± 0.84	1.73 ± 0.25	1.23 ± 0.27
Linalool^4^	0.14 ± 0.01	0.11 ± 0.01	0.15 ± 0.01 *	0.08 ± 0.01
Lilac aldehyde A^4^	36.63 ± 4.34 *	80.64 ± 8.02	8.41 ± 1.21	19.34 ± 3.83
Lilac aldehyde B^4^	61.92 ± 7.02 *	122.04 ± 12.12	13.81 ± 1.88	24.89 ± 5.13
Lilac aldehyde C^4^/Benzyl acetate	7.29 ± 2.09 *	11.99 ± 2.43	1.37 ± 0.21	2.20 ± 0.40
Lilac alcohol^3^	2.15 ± 0.25 *	4.05 ± 0.38	0.58 ± 0.07	1.01 ± 0.18
				
**Sesquiterpenes**^2^	**0.05%**	**0.01%**	**0.09%**	**0.02%**
B-Farnesene	0.11 ± 0.02 *	0.05 ± 0.01	0.07 ± 0.02	0.03 ± 0.00
				
**Irregular terpenes**^2^	**0.05%**	**0.02%**	**0.12%**	**0.04%**
6-Methyl-5-hepten-2-one	0.11 ± 0.02	0.10 ± 0.02	0.10 ± 0.01	0.06 ± 0.01
				
**Unidentified with Kovat's retention index (*R*_*I*_)^2^**	**3.61%**	**3.35%**	**8.11%**	**2.95%**
Unknown 1 (978)	0.99 ± 0.10	0.95 ± 0.12	0.90 ± 0.08 *	0.65 ± 0.08
Unknown 2 (992)	3.63 ± 0.41 *	2.47 ± 0.55	4.98 ± 0.66 *	1.86 ± 0.23
Unknown 3 (1009)	0.09 ± 0.01	0.05 ± 0.01	0.09 ± 0.02 *	0.02 ± 0.01
unknown 4 (1112)	0.29 ± 0.06 *	0.82 ± 0.11	0.14 ± 0.02 *	0.26 ± 0.04
unknown 5 (1191)	2.33 ± 0.36 *	9.85 ± 1.20	0.51 ± 0.06 *	1.17 ± 0.19

^1 ^Compounds within a chemical class ordered according to retention time.

^2^The relative contribution of each chemical class is given in bold (in %).

^3^Electrophysiologically (GC-EAD) and ^4^behaviorally active odor compounds in *Hadena bicruris *as described by Dötterl *et al*. (2005, 2006) and personal communication [17,20].

### Morphology

Flower number was higher in male plants compared to female plants in both populations (flowers plant^-1 ^± SE: Switzerland males 7.74 ± 0.53, Switzerland females 5.26 ± 0.50, Mann-Whitney U-test: U = 2887, P < 0.001; Spain males 8.63 ± 0.54, Spain females 4.67 ± 0.19, Mann-Whitney U-test: U = 8908, P < 0.001). Flower diameter was significantly smaller in male flowers than in female flowers in both populations (mean flower diameter ± SE: Switzerland males 2.51 ± 0.03, Switzerland females 2.60 ± 0.03, t-test: t = 2.017, df = 193, P < 0.05; Spain males 2.67 ± 0.02, Spain females 2.84 ± 0.02, t = 5.405, df = 351, P < 0.001).

### Moth behavior

Most flower-naïve pollinator moths chose male flowers in their first approach to *S. latifolia*, suggesting higher attractiveness of male flowers compared to female flowers, however the preference was only significant for male moths. Of the 25 male moths tested, 6 chose female and 19 male flowers (Chi^2 ^= 6.76, df = 1, P = 0.009). Of the 31 unmated females tested, 11 chose female and 20 male flowers (Chi^2 ^= 2.61, df = 1, P = 0.11).

## Discussion

Floral traits that increase pollinator attraction are expected to evolve under stronger pollinator-mediated selection in male plants because males compete for pollinator visitation whereas females are usually limited by resources other than pollen [1,3-5]. For female plants, and especially in nursery pollination systems, attractiveness to pollinators is risky because it is linked to seed predation [11,12]. Consistent with theoretical expectations of sexual selection on floral attractiveness, we found that individual male flowers were more attractive to naïve pollinators, especially to male moths. The likely reason for this is the significantly higher emission of floral scent per flower in male versus female flowers.

In many dioecious plant species, males produce larger or more flowers than females [3,10,22-24]. Plants with increased floral display usually receive higher numbers of visits by pollinators [25-28]. In *S. latifolia*, male flowers are smaller than female flowers, but male plants produce more flowers than females, suggesting a trade-off between flower size and number [29]. *Silene latifolia *pollinators prefer plants with larger floral displays [13]. Therefore, the increased number of flowers found on male *S. latifolia *plants enhances the attractiveness to pollinators [29,30]. However, as yet it was not clear whether the increased attractiveness of male plants is simply a function of the higher number of flowers, or whether individual flowers have evolved traits of higher attractiveness to pollinators. We showed in dual choice experiments in the wind-tunnel that individual male flowers are indeed more attractive to the males of the main pollinator moth. Male flowers, despite being smaller than female flowers, produced significantly higher amounts of odor, both in the Swiss and Spanish population. The significant interaction between sex and population indicates that the sex differences in the amount of scent produced is different among the two populations. Indeed, in the Spanish population, the sex difference was less pronounced, and these plants emitted an overall lower amount of scent. Population specific differences in sex specific traits may be related to differences in pollinator composition, or differences in Swiss and Spanish *Hadena *moths, however, data are not available.

Interestingly, we found that in both populations more of the compounds involved in pollinator attraction, so-called active compounds, were significantly different among sexes, suggesting that selection for higher attractiveness is mediated by the sensory ecology of the pollinator. Overall, our results strongly suggest that in *S. latifolia*, scent is more important for the attractiveness of individual flowers than size, as we used flowers of similar size in our behavioral assays. Other studies on *S. latifolia *floral scent emission failed to detect a statistically significant higher odor production by males [20], but similar trends were found [31]. Both studies, however, were not designed to investigate the effect of gender on floral fragrance and analyzed fewer plants than our study; given the usually high variation in floral scent, large sample size is an important factor in detecting significant effects. Sex differences in amounts of scent were also detected in other plant species, with male plants releasing more attractive volatiles or higher amounts than conspecific females [32,33]. Ashmann et al. (2005) showed that in the gynodioecious wild strawberry *Fragaria virginiana *the smaller hermaphroditic flowers emitted significantly more odor, which resulted in more visits by pollinators compared to conspecific females [34]. However, these authors suggest that the odor of hermaphroditic flowers is preferred due to the production of unique compounds produced by the anthers, rather than due to quantitative differences in odor production.

A positive association between scent concentration and attractiveness was also found in earlier experiments, where higher odor concentration amplified the response of the pollinators in wind tunnel bioassays with *Hadena bicruris *[17]. Schiestl (2004) showed that larger amounts of a biologically active floral odor compound attracted more pollinators of the orchid *Chiligottis trapeziformis *[35]. In natural populations of *S. latifolia*, preferences of pollinators for male flowers could be the result of learning, since male flowers produce higher sugar concentrations and thus higher quality rewards [13]. As we used naïve pollinators for our experiments, learning should not have influence the preferred choice of male flowers. Alternatively, preference of stronger odor sources may be due to the stronger excitement of olfactory receptors, a form of supernormal stimulation found in floral mimicry systems [35].

Interestingly, the higher attractiveness of male flowers was less pronounced in female moths, which showed no significant preference for male flowers. Female moths need to find flowers for oviposition, besides nectar consumption for energy supply. Because only female flowers are optimal sites for larval development, female moths are expected to evolve preference for female flowers, and especially so after mating. It would be interesting to test in future experiments behavioral preferences of unmated vs. mated females. This situation represents an interesting example of sexual conflict, both in the plant and its pollinator [36]. In the moths, male preference of stronger odor emission acts against female interests, i.e. preference of female flowers, at least after mating. In the plant, the stronger odor emission for increased pollen export should be selected against in female plants that are probably not limited in reproductive success by pollen income and should avoid the attraction of seed predating female moths.

## Conclusion

In conclusion our study confirms the predictions by sexual selection theory that male flowers should be more attractive to pollinators than female flowers. This result was not obvious in this plant species, as male are smaller than female flowers, however, the floral scent is decisive for attractiveness in this pollination system. We suggest that taking into account the pollinators' sensory and behavioral ecology will likely give a better picture on sexual and natural selection acting on floral signals, and thus lead to a better understanding how flowers evolve under pollinator-mediated selection.

## Methods

### (a) Plant material and odor collection

For volatile collection, *S. latifolia *plants from seeds collected in two populations (Leuk, Switzerland, n = 25; Ribes de Fraser, Spain, n = 4) were grown in a common garden setup in a green house. Floral odor of 555 *S. latifolia *plants (Switzerland: 79 males and 123 females, Spain: 217 males and 136 females) was collected in May at night, between 9 p.m. and 7 a.m. by headspace sorption as described in [18]. We used only newly opened flowers for odor collection. All floral odor samples were stored in sealed glass vials at -20°C for subsequent gas chromatograph (GC) analysis.

### (b) Chemical analysis

The samples were analyzed with a gas chromatograph (GC, Agilent 6890 N) fitted with a HP5 column (30 m × 0.32 mm internal diameter × 0.25 μm film thickness) and a flame ionization detector (FID); hydrogen served as carrier gas. We injected one micro-liter of each odor sample splitless at 50°C (1 min) followed by heating to 150°C at a rate of 5°C min^-1^, and then to 300°C at a rate of 10°C per minute before keeping the oven at 300°C for ten minutes. For odor compound identification peak retention times were compared with those of authentic standard compounds and confirmed by comparison of spectra obtained by gas chromatography-mass spectrometry (GC-MS). One micro liter aliquots of the odor samples were injected into a GC (HP G1800A) with a mass selective detector using the oven and column parameters described above. We discriminated between active and non-active compounds in the attraction of *Handena bicruris *on the basis of electrophysiological recordings and behavioral assays done in another study [20].

### (c) Morphology

We counted all newly opened flowers per plant before odor sampling. The next morning, two flowers per plant were collected and images taken with a digital camera. We measured floral diameter from these images using the software ImageJ  and calculated average values.

### (d) Moth rearing

*Hadena bicruris *(Lepidoptera: Noctuidae) moths were bred in the lab, starting with adult moths (n = 10, 50% males and 50% females) collected in the surroundings of Zürich, Switzerland, during summer 2005. Wild moths (approx 10 per year) were added to the colony each summer in order to ensure outbreeding. All insects were reared in controlled conditions under a L16:D8 photoperiod and temperature of 20 ± 2°C, at 65 – 80% relative humidity (RH). After hatching of the eggs, larvae were kept in separate plastic containers to avoid cannibalism and were individually fed with fresh capsules of *Silene latifolia *until pupation. The pupae were then sexed and placed in separate rearing cages until emergence. These emerged adults were used for behavioral experiments before they had any contact to flowers. Over three successive seasons, a total of 56 flower-naïve moths (31 females and 25 males) were used for dual choice experiments with flowers of *S. latifolia*.

### (e) Behavioral assays

A 200 × 80 × 80 cm wind tunnel was used for behavioral tests. A Fischbach speed-controller fan (D340/E1, FDR32, Neunkirchen, Germany) pushed air through the tunnel with an air velocity of 0.35 m s^-1^. Four charcoal filters (145·457 mm, carbon thickness 16 mm, Camfil Farr, Reinfeld, Germany) cleaned the incoming air. The experiments were performed at night with red light illumination (< 0.01 μE) 1–3 h after the start of the dark period. An hour prior to testing, the moths were exposed to ambient room temperature (20°C), RH (65%) and experimental light conditions. Naïve moths were tested individually in the wind tunnel. Each moth was placed in an open glass tube mounted on a stand at the downwind side of the tunnel. At the upwind end were set two freshly collected flowers of *S. latifolia *(from the Swiss population) of different sexes and approximately equal corolla size (to avoid an effect of flower size). A distance of 20 cm separated the two flowers in order to ensure that the moth's response entirely depended on scent source. After take-off, each moth was followed visually until it landed on one of the two flowers, which always resulted in proboscis extension and nectar drinking.

### (f) Statistical analysis

All data were tested for homogeneity of variances (Levene's test) and for normality (Kolmogorov-Smirnov test). We used a GLM approach to examine the effects of sex and population on mean absolute odor emission. Log-transformed total amount of odor was used as dependent variable and sex and population as factors. Floral diameter was used as covariate to correct for flower size. Nonparametric Mann-Whitney U-tests were used to analyze the differences in absolute amounts of individual compounds and of flower numbers between males and females in each population separately, since no transformation allowed analysis with a parametric test. Flower diameter was analyzed by a t-test. Frequency of moth choices were compared using a Chi^2 ^test. All analyses were carried out using SPSS 11.0.4 for Mac OS X (SPSS Inc., Chicago, USA).

## Authors' contributions

MOW collected and analyzed data, and wrote part of the manuscript, PAP performed bioassays with moths, AW designed the experiments, FPS designed experiments, analyzed data, and wrote part of the manuscript. All authors contributed to the discussion, read and approved the final manuscript.
